# Study of Arachidonic Acid Pathway in Human Bladder Tumor

**DOI:** 10.4137/sart.s2151

**Published:** 2009-12-09

**Authors:** Masahide Matsuyama, Rikio Yoshimura

**Affiliations:** Department of Urology, Osaka City University Graduate School of Medicine, 1-4-3 Asahi-machi, Abeno-ku, Osaka, 545-8585, Japan.

**Keywords:** cyclooxygenase, lipoxygenase, peroxisome proliferator activator-receptor-γ, bladder tumor

## Abstract

Recent epidemiological studies and animal experiments have demonstrated that nonsteroidal anti-inflammatory drugs (NSAIDs) reduce the incidence of colorectal carcinoma. Cyclooxygenase (COX) is the principal target of NSAIDs. COX is the first oxidase in the process of prostaglandin production from arachidonic acid. COX enzyme may be involved in the initiation and/or the promotion of tumorigenesis due to NSAIDs inhibition of COX. Lipoxygenase (LOX) is also an initial enzyme in the pathway for producing leukotrienes from arachidonic acid. Similar to COX, LOX enzyme may also be involved in the initiation and/or promotion of tumorigenesis. Peroxisome proliferator activator-receptor (PPAR)-γ is a ligand-activated transcriptional factor belonging to the steroid receptor superfamily. PPAR-γ plays a role in both adipocyte differentiation and tumorigenesis. PPAR-γ is one target for cell growth modulation of NSAIDs. In this review, we report the expression of COX-2, LOX and PPAR-γ in human bladder tumor tissues as well as the effects of COX-2 and LOX inhibitors and PPAR-γ ligand.

## Introduction

The natural history of the bladder tumor (BT) is not well understood, but exposure to carcinogens, including aromatic amines, is considered a major risk factors for the development of BT. Workers exposed to aromatic amines frequently have a mutated p53 gene, a tumor suppressor gene involved in the tumorigenesis of many tumors.

Non-steroidal anti-inflammatory drugs (NSAIDs) have anti-tumor effects for the RCC patient, thus, attracting a great deal of attention. The typical target of NSAIDs is cyclooxygenase (COX). In recent reports, a number of patients have had significantly low risks of colorectal cancer while they continued using NSAIDs typified by aspirin. Consequently, the suppression of tumorigenesis by administering NSAIDs has come into focus. It was also reported that the size and number of adenoma were markedly reduced when sulindac, a type of NSAIDs was given to patients with familial adenomatous polyposis, a high risk group for colorectal cancer.^1^

It is known that NSAIDs inhibit the activity of COX and production of prostaglandin. NSAIDs also stimulate peroxisome proliferator activator-receptor (PPAR)-γ and inhibit the production of chemical mediators such as tumor necrosis factor-α, interleukin-1β and interleukin-6 through the expression of PPAR-γ in leukocytes. PPAR-γ is thus a promising target for cell growth modulation by NSAIDs.

In this review, we discuss the possibility that the target of arachidonic acid pathway metabolite may be a new anti-tumor strategy for human BT.

## Arachidonic Acid Pathway

The metabolism of arachidonic acid by either COX pathway or lipoxygenase (LOX) pathway generates eicosanoids, which have been implicated in the pathogenesis of a variety of human diseases, including cancer, and are considered important in tumor promotion, progression, and metastasis.^2^

COX is the first enzyme in the pathway for producing prostaglandin (PG) and thromboxane (Tx) from arachidonic acid, and can occur as three isoforms, COX-1, COX-2 and COX-3. The enzymes of both COX-1 and COX-2 are transformed from the cell membrane phospholipid to arachidonic acid by the phospholipase A_2_, and then transform arachidonic acid to PGH_2_ through PGG_2_. COX-1 occurs in tissues and cells and works to protect the cell. COX-2 express momentarily and strongly in response to growth factors and some endotoxins. It is involved with inflammation, cell proliferation and differentiation. 3 Recently, COX-2 has also been shown to play an important role in tumorigenesis.^1^ Although the existence of COX-3 has recently been reported, it continues to be argued. In pathogenesis of BT, Both COX-2 and PGs (especially PGE_2_) play a role in development of BT.

LOX is the first enzyme in the pathway for producing leukotriene (LT) from arachidonic acid. Isoenzymes of LOX include 5-LOX, 12-LOX and two 15-LOX isoforms (15-LOX-1, 15-LOX-2). These catalyze the biosynthesis of biologically active compounds such as LTs and hydroxyeicosatetraenoic acids (HETEs).^4^,^5^ 5-LOX catalyzes the first step in oxygenation of arachidonic acid to produce 5-hydroperoxyeicosatetraenoic acid (5-HPETE), and the subsequent metabolism of 5-HPETE to 5-HETE and LTs. LTs belong to an important group of pro-inflammatory mediators that are synthesized from arachidonic acid via the 5-LOX pathway. The activity of 5-LOX leads to the formation of unstable LTA_4_, which can be converted into either LTB_4_, or cysteinyl LTs (LTC_4_, LTD_4_ and LTE_4_).^6^

The 12-LOX, includes platelet 12-LOX, and leukocyte 12-LOX that oxygenate arachidonic acid at position C-12 to produce 12-hydroperoxyeicosatetra-enoic acid and then 12-HETE.^2^ Whereas 5-LOX, 12-LOX and 15-LOX-1, have pro-tumorigenic roles, 15-LOX-2 appears to have an anti-tumorigenic roles. The administration of LOX inhibitor may inhibit matrix metalloproteinase (MMP)-2, it may induce down regulation such as BCL-2 and nuclear factor-kappaB (NF-λB), and it may activate such as PPAR-γ, apoptosis activating factor-1 and caspase-3.

## PPAR

PPARs are members of the nuclear receptor super-family of ligand-activated transcriptional factor such as steroids, thyroid hormone, vitamin D_3_ and retinoic acid.^7^ PPAR binds to peroxisome proliferator response element (PPRE) as a heterodimer with the retinoic receptor (RXR) in the regulation of PPAR target genes. PPARs may be important immunomodulatory factors as well as fatty acid regulators. PPARs modulate these activities in different immune cell types such as monocyte/macrophages, lymphocytes, and endothelial cells.

Three PPAR subtypes (α, β, and-γ) have been identified. PPAR-α is highly expressed in the liver, heart, kidney, muscle, brown adipose tissue, and gut tissues which exhibit high carbolic rates towards fatty acid.^8^ PPAR-β is expressed ubiquitously, and its function is relatively unknown.^9^ Recent studies suggest that PPAR-β may be a target for NSAID-induced tumor suppression in colorectal tumors. PPAR-γ is expressed at high-level in adipose tissue and is a critical regular of adipocyte differentiation. In addition, PPAR-α, and –γ may be important immunomodulatory factors. PPAR-α-knockout mice exhibit exacerbated inflammatory responses, and LTB_4_, a chemotactic mediator, seems to regulate the clearance of itself as an agonist of PPAR-α. PPAR-γ is also expressed in the immune system tissues and cells (spleen, bone-marrow, monocytes, helper T-cell clones) and skeletal tissues (bone, synovioum, chondrocytes). 8 PPAR-γ also have an anti-tumorigenic roles.

Recent data have shown that PPAR-γ ligands lead to inhibition of phorbol ester-induced nitric oxide and macrophage-derived cytokines. PPAR-γ ligands also induce apoptosis in macrophage, fibroblasts, and endothelial cells.^10^ PPAR-γ plays a role in both adipocyte differentiation and tumorigenesis. PPAR-γ ligands lead to inhibition of the expression of nitric oxide, cytokines, chemokines and adhesion molecules, in part by antagonizing the activities of transcriptional factors. Furthermore, PPAR-γ ligands including anti-diabetic thiazolidinedione (such as troglitazone) and 15-deoxy-Δ^12^,^14^ -prostaglandin J_2_ (15-d-PGJ_2_) have potent tumor modulatory effects on several tumors.^11^,^12^

15-d-PGJ_2_ induces apoptosis in macropharge, endothelial cell, and choriocarcinoma cell^13^–^15^ as well as thiazolidinediones-induced fibroblast apoptosis.^16^ PPAR-γ ligands also inhibit vascular endothelial cell growth factor-induced angiogenesis *in vivo.*^17^ Angiogenesis is important for carcinogenesis. Anti-angiogenetic therapy is highly promising since it does not induce aquired anticancer drug resistance.^18^,^19^ Drevs et al. demonstrated the effect of PTK787/ZK 222584, a specific inhibitor of vascular endothelial growth factor receptor tyrosine kinases, on primary tumor, metastasis, vessel density, and blood flow in an animal model of renal cell carcinoma.^20^ PPAR-γ agonists induce apoptosis in endothelial cells and inhibit vascular endothelial growth factor-induced angiogenesis in rats. Therefore, PPAR-γ ligands may have anticancer-effects through inhibition of cell proliferation and angiogenesis.

## COX and BT

**Figure 1:**
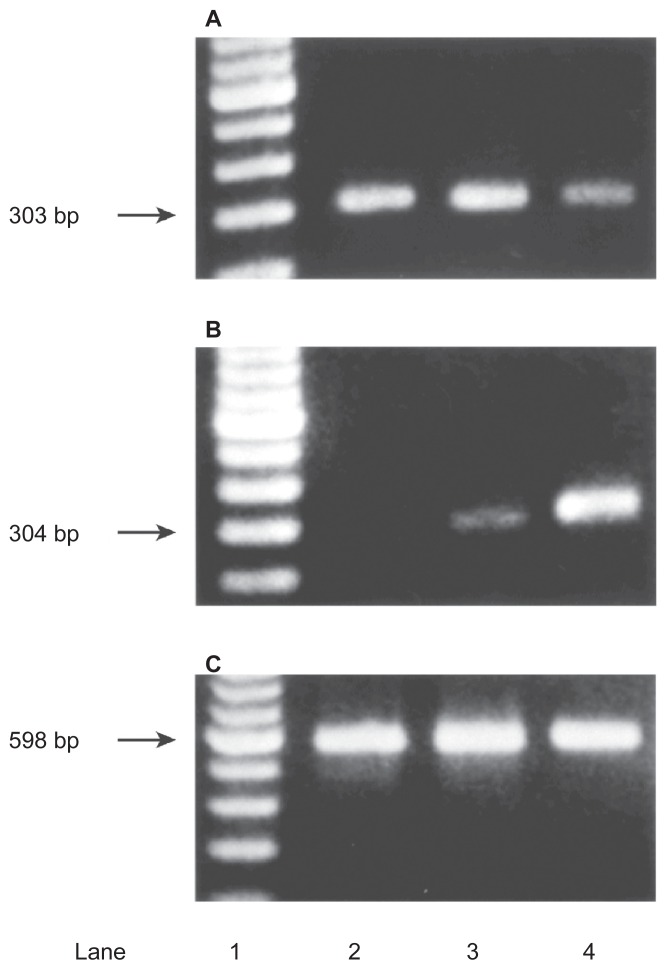
RT-PCR analysis of COX-1 and COX-2 in bladder tissues Total RNA was purified from 10 BT, 5 chronic cystitis and 5 normal bladder patients. Primer for COX-1, COX-2, and G3PDH generated the expected 303, 304, and 598 nucleotide bands after cycles of PCR. Specific band of COX-1 protein was detected in all samples (**A**, lane 2, 3, 4). However, specific bands of COX-2 protein was detected in samples from BT (B, lane 4), while samples from chronic cystitis displayed a very weak band (**B**, lane 3), and samples from normal bladder displayed no clear band (**B**, lane 2) in the study. **C**) G3PDH.

COX-2 expression in BT tissues was stronger than in chronic cystitis (CC) and normal bladder (NB) tissues by immunohistochemical staining (118 BT, 10 CC and 8 NB tissues) or RT-PCR (Fig. 1). We classified 3 categories (epithelium, blood vessel, stromal tissue) in BT tissues, and examined them for intensity of COX-2 expression. COX-2 expression was more intensive and extensive in all categories of BT tissues than CC and NB tissues. Significant differences occurred between grades of BT tissues in only epithelium in COX-2 expression. COX-2 expression was stronger in G3 cancer and advanced-stage BT (pT2 or above).^21^

Mohammed et al. reported the expression of COX-1 in both BT and NB tissues by immunohistochemical staining and western blot. COX-2 was stronger in the advanced stage BT than NB tissue.^22^ Komhoff et al. reported COX-2 expression was stronger in low differentiation BT by immunohistochemical staining.^23^ Shirahara et al. reported COX-2 expression was stronger in the advanced-stage BT, and COX-2 expression was most strongest in the highest malignancy of BT in situ, and COX-1 expression was nothing in any BT tissues by immunoblot and immunohistochemical staining.^24^ Margulis et al. reported COX-2 was not expressed in NB urothelium. COX-2 over expression is associated with pathological and molecular features of biologically aggressive disease, suggesting a role for COX-2 in BT development and invasion.^25^

Okajima et al. reported the administration of nimesulide and piroxicam as selective COX-2 inhibitor could reduce remarkable lowering of tumorigenesis rate in the BT using rat model which superficial BT initiating the tumor by administering the N-butyl-N-nitrosoamine.^26^ Okamoto et al. reported etodolac (selective COX-2 inhibitor) exhibited anti-tumor activity and induced E-cadherin expression in BT cells and might be useful for the clinical treatment and prevention of BT, especially in poorly differentiated bladder cancer with high COX-2 and low E-cadherin expression.^27^

However, recently accepted explanation is that only celecoxib, among the COX-2 inhibitors induces apoptosis of cancer cells. We suggest the increased apoptosis cells produced by celecoxib might be associated with decreased PGE_2_ production.

Generally, Bcl-2 is a key factor suppressing the apoptosis accompanying of caspase. Gee et al. reported treatment with 100 μM celecoxib resulted in significant apoptosis in three kinds of BT cell lines, which was associated with down-regulation of Bcl-2.^28^

In our experiment, we could not demonstrate significant apoptosis induction by administering eight kinds of COX-2 inhibitors (10–80 μM) into the human BT cell lines (T24) (Table 1). Additionally, we could not demonstrate the significance of both selective COX-2 inhibitor (etodolac, meloxicam, nimesulide and NS398) and relatively poor selective COX-2 inhibitor (ibuprofen, indomethacin, piroxicam and s-naparoxen).^29^

In conclusion, COX-2 expression is strong in BT, but the anti-tumor effect of COX-2 inhibitor is very weak in BT patients in a single administration at a clinical dose. It may be difficult to suppress the growth of BT for chemotherapy. COX-2 inhibitor is suitable for chemopreventive therapy.

## LOX and BT

**Figure 2:**
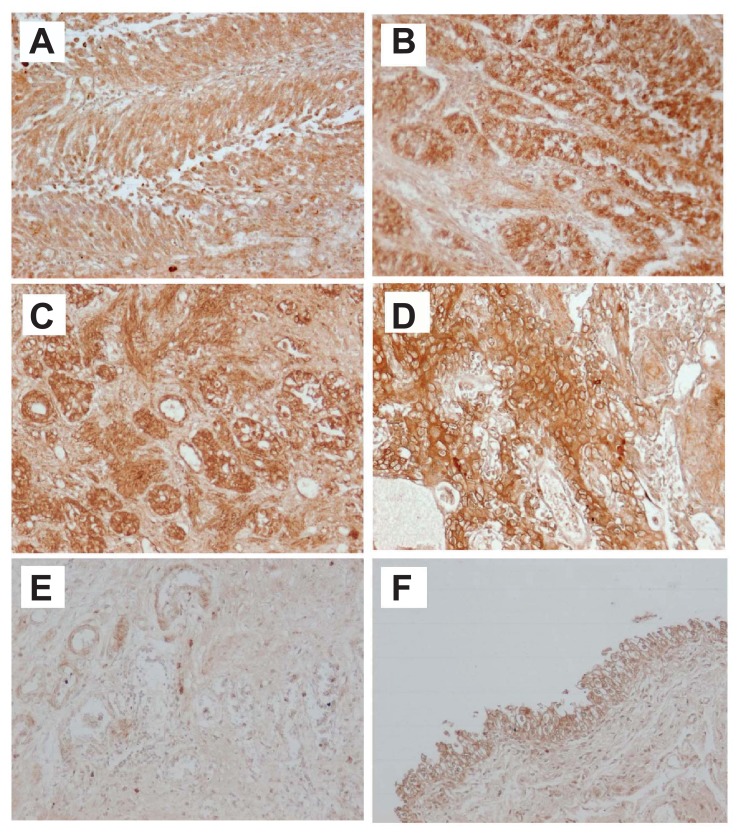
5-LOX immunostaining in bladder tissues Very weak expression of 5-LOX was detected in chronic cystitis **E**) and normal bladder tissues. **F**) In contrast, 5-LOX was strongly expressed in BT tissues, transitional cell carcinoma-G1, -G2 and –G3 (**A**–**C**) and squamous cell carcinoma **D**) tissues.

We have shown that 5- and 12-LOX expressions in BT tissues were stronger than those in NB tissues by immunohistochemical staining (170 BT, 20 CC and 20 NB tissues) (Fig. 2) and RT-PCR. We classified 3 categories (epithelium, blood vessel, stromal tissue) in BT, CB and NB tissues, and examined the intensity of 5- and 12-LOX expressions. 5- and 12-LOX expressions were more intensive and extensive in all categories of BT tissues as compared with CC and NB tissues (Table 2). 5-LOX expression was stronger in G3 cancer and advanced-stage BT (pT2 or above) than G1 cancer and early-stage BT (pT1 or below).^2^

**Figure 3:**
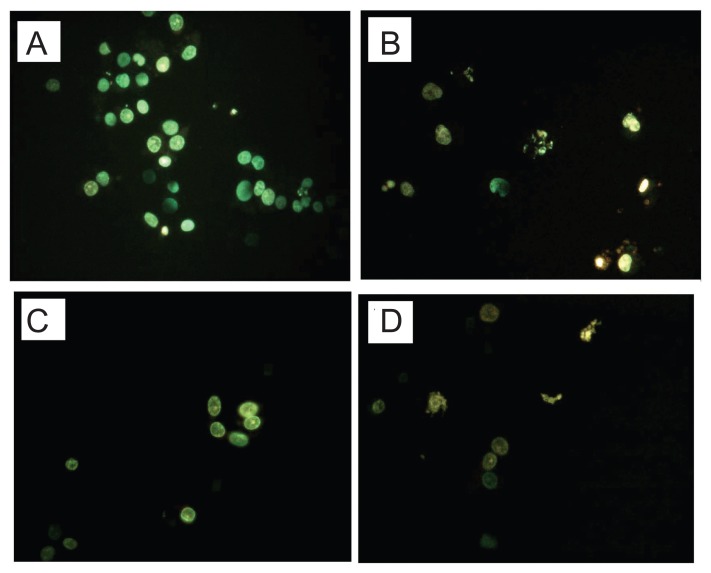
Effects of LOX inhibitor in induction of apoptosis on BT cells Human BT cell line (T24) treated with 5-LOX inhibitor caffeic acid **B**), and non-specific LOX inhibitor NDGA **D**) showed chromatin condensation, cellular shrinkage, small membrane-bound bodies (apoptotic bodies), and cytoplasmic condensation. Cells with 12-LOX inhibitor baicalein only slightly showed the same apoptotic changes **C**). In contrast, untreated cells **A**) maintained normal chromatin patterns and cell size.

At 10–80 μM, some, but not all LOX inhibitors reduced the viability of BT cell lines (T24) by MTT assay (Table 1). 5-LOX inhibitor appeared more potent than the 12-LOX inhibitor.^30^ BT cells treated with some LOX inhibitor (50 μM) showed chromatin condensation, cellular shrinkage, apoptotic bodies, and cytoplasmic condensation by hoechest staining (Fig. 3).

BT cells treated with 5-LOX inhibitor (100 μM) also entered early apoptosis, but not late apoptosis, necrosis or DNA fragmentation by flow cytometry.

Hayashi et al. reported BT cells frequently expressed 5-LOX. 5-LOX inhibitor (AA861) revealed the strongest growth suppression of those cells compared to other LOX and COX pathway inhibitors, and the growth suppression effects were considered to be due to inhibition of the enzymatic activity.^31^

In conclusion, 5-LOX expression is strong in BT, especially low differentiation and advanced-stage BT. The anti-tumor effect of 5-LOX inhibitor is significantly stronger than those of COX-2 inhibitor. The anti-tumor effect of 5-LOX inhibitor is weak in BT patients in a single administration at a clinical dose. 5-LOX inhibitor is suitable for chemopreventative therapy, like COX-2 inhibitor.

## PPAR-γ and BT

By immunohistochemical staining and RT-PCR, PPAR-γ expression is increased in BT tissues as compared with NB tissues (170 BT and 20 NB tissues). In all categories (epithelium, blood vessel, stromal tissues) of BT and NB tissues, the intensity of PPAR-α, -β expression was not significantly different. In all categories, PPAR-γ expression was significantly greater in BT tissues than in NB tissues. Only epithelium, PPAR-γ expression was stronger in G3 cancer. In all categories, PPAR-γ expression was stronger in advanced-stage BT (pT2 or above).^32^

**Figure 4:**
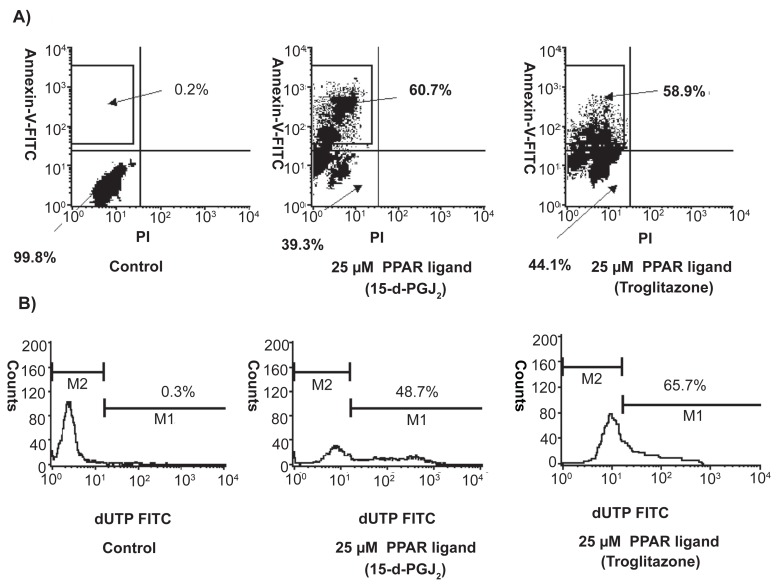
Effects of PPAR-γ ligands on apoptosis by flow cytometry in BT cells Treatment with 25 μM PPAR-γ ligands (troglitazone and 15-d-PGJ_2_) induced early apoptosis not late apoptosis or necrosis in BT cell line (T24). The top left quadrants represent early apoptosis. Diagrams of FITC-Annexin V/PI flow cytometry are presented. **A**) Treatment with 25 μM PPAR-γ ligands (troglitazone and 15-d-PGJ_2_) could induce DNA fragmentation in BT cell line (T24). Typical flow cytometry analysis histograms are presented **B**).

At 10–40 μM, PPAR-γ ligands (troglitazone and 15-d-PGJ_2_) reduced the viability of BT cell lines (T24) by MTT assay (Table 1). BT cells treated with PPAR-γ ligands (25 μM troglitazone and 15-d-PGJ_2_) could early apoptosis, not late apoptosis or necrosis and DNA fragmentation by flow cytometry (Fig. 4). PPAR-γ ligands (20 μM troglitazone and 15-d-PGJ_2_) also induced apoptosis in BT cells by hoechest staining.^33^

About anti-tumor mechanism of troglitazone and 15-d-PGJ_2_, Chaffer et al. reported troglitazone induced G0/G1 growth arrest while 15-d-PGJ_2_ induced apoptosis. Troglitazone and 15-d-PGJ_2_ inhibit growth of BT cell lines through different mechanisms and the effects of both agents are PPAR-γ independent.^34^

Bacillus Calmette-Guérin (BCG) is considered to be one of the most effective treatments for superficial and in situ BT. Lodillinsky et al. reported BCG induced functional PPAR-γ in BT cells in vivo and in vitro, being these receptors intrinsically involved in the anti-tumor activity of BCG.^35^

In conclusion, PPAR-γ is strong in BT, and the anti-tumor effect of PPAR-γ ligand is significantly greater than that of 5-LOX inhibitor. The anti-tumor effect of PPAR-γ ligand is relatively weak. Targeting PPAR-γ in BT is likely to be useful more as a chemopreventive rather than chemotherapeutic.

## Conclusions

There is no discussion that COX-2, LOX (especially, 5-LOX) and PPAR-γ are involved in the initiation and promotion of BT tissues. It may be possible to use COX-2 and 5-LOX inhibitor, and PPAR-γ ligand as an anti-tumor drug for chemopreventive therapy in a single administration at a clinical dose. However, it may be difficult to use the COX-2 and 5-LOX inhibitor, even if PPAR-γ ligand in single administration at a clinical dose in expectation for chemotherapeutic therapy. However, the clinical application of PPAR-γ ligand and 5-LOX inhibitor require further research and consideration, the target of PPAR-γ ligand and 5-LOX inhibitor is a novel strategy over human BT.

We conclude the administration of COX-2 and 5-LOX inhibitor, and PPAR-γ ligand are useful with usual treatment in human BT. In near future, the combination therapy of COX-2 and 5-LOX inhibitor, and PPAR-γ ligand will be useful new treatment of human BT.

## Figures and Tables

**Table 1:** Effects of COX and LOX inhibitors, and PPAR-γ ligand in viability of human BT cell line (T24).

	5 μM	10 μM	20 μM	40 μM	80 μM
***COX-2 inhibitor***					
*Selective COX-2 inhibitor*					
Etodolac	not shown	98.7%	105.9%	100.7%	94.1%
Meloxicam	not shown	115.2%	97.7%	99.1%	72.6%
Nimesulide	not shown	130.6%	122.2%	114.9%	91.5%
NS398	not shown	86.1%	96.1%	77.4%	75.9%
*Poor selective COX-2 inhibitor*					
Ibuprofen	not shown	112.6%	113.7%	94.9%	97.4%
Indometacin	not shown	130.3%	127.1%	113.5%	122.0%
Piroxicam	not shown	109.3%	101.4%	91.0%	81.1%
S-naproxen	not shown	95.5%	101.0%	102.5%	101.3%
***LOX inhibitor***					
5-LOX inhibitor (Caffeic acid)	not shown	90.1%	89.7%	29.8%	9.9%
12-LOX inhibitor (Baicalein)	not shown	98.2%	99.0%	92.5%	62.8%
Non selective LOX inhibitor (NDGA)	not shown	95.8%	63.1%	23.3%	13.2%
***PPAR-***γ ***ligand***					
Troglitazone	62.7%	52.2%	31.8%	24.8%	not shown
15-d-PGJ_2_	88.3%	71.0%	19.5%	8.1%	not shown

The dose-response analysis of viability in human BT cell line (T24) treated with COX-2 and LOX inhibitors, and PPAR-γ ligand (5–80 μM, 48 hr) was measured by the MTT assay and expressed as % of control culture conditions.

**Table 2:** Statistical analysis of 5- and 12-LOX immunostaining.

Tumor type	Av. ± SD		
	
	Epithelium	Blood vessel	Stromal tissue
***5-LOX***			
Transitional cell Ca.			
Grade 1	2.6 ± 0.8*	2.3 ± 0.8*	2.4 ± 1.2*
Grade 2	3.0 ± 1.1*	2.5 ± 1.1*	2.3 ± 1.4*
Grade 3	3.6 ± 1.1*	2.6 ± 1.3*	2.6 ± 0.9*
Early stage	2.0 ± 1.2*	2.5 ± 0.9*	2.3 ± 1.2*
Advanced stage	3.3 ± 1.3*	2.7 ± 1.3*	2.6 ± 1.1*
Squamous cell Ca.	3.5 ± 0.5*	3.5 ± 0.5*	3.5 ± 0.5*
Chronic cystitis	0.6 ± 0.5	0.4 ± 0.4	0.4 ± 0.4
Normal bladder tissue	0.8 ± 0.6	0.7 ± 0.5	0.6 ± 0.5
***12-LOX***			
Transitional cell Ca.			
Grade 1	1.8 ± 0.6*	1.7 ± 0.8*	1.7 ± 0.8*
Grade 2	1.7 ± 0.8*	1.7 ± 0.8*	1.8 ± 1.0*
Grade 3	2.0 ± 1.1*	2.1 ± 1.0*	1.9 ± 1.0*
Early stage	1.9 ± 1.0*	1.7 ± 0.9*	1.7 ± 1.2*
Advanced stage	2.1 ± 0.9*	1.8 ± 1.0*	1.9 ± 1.3*
Squamous cell Ca.	2.0 ± 0.5*	2.2 ± 0.5*	1.8 ± 0.5*
Chronic cystitis	0.7 ± 0.5	0.5 ± 0.5	0.4 ± 0.4
Normal bladder tissue	0.5 ± 0.4	0.4 ± 0.3	0.4 ± 0.3

**Notes:** Graded 0 to 4 on the coded sections by two observers in a blinded manner. 0, no staining; 4, maximum intensity. Statistical analysis was performed using the analysis of variance.

* p < 0.001.
